# Stroke Risk among Patients with Type 2 Diabetes Mellitus in Zhejiang: A Population-Based Prospective Study in China

**DOI:** 10.1155/2016/6380620

**Published:** 2016-06-14

**Authors:** Lihua Guo, Min Yu, Jieming Zhong, Haibin Wu, Jin Pan, Weiwei Gong, Meng Wang, Fangrong Fei, Ruying Hu

**Affiliations:** Zhejiang Provincial Center for Disease Control and Prevention, 3399 Binsheng Road, Hangzhou 310051, China

## Abstract

*Objective*. This study aimed to explore the incidence of stroke and stroke subtypes among patients with type 2 diabetes mellitus (T2DM) based on the long-term surveillance data in Zhejiang, China, during 2007 to 2013.* Materials and Methods*. During January 1, 2007, and December 31, 2013, a total of 327,268 T2DM and 307,984 stroke patients were registered on Diabetes and Stroke Surveillance System, respectively. Stroke subtypes were classified according to standard definitions of subarachnoid hemorrhage, intracerebral hemorrhage, and ischemic stroke. The incidence of stroke and stroke subtypes was calculated by standardized incidence ratio (SIRs) with 95% confidence intervals (CIs) compared with general population.* Results*. The incidence of stroke and stroke subtypes among patients with T2DM was significantly higher than in general population. Stroke risk was found significantly increased with an SIR of 3.87 (95% CI 3.76–3.99) and 3.38 (95% CI 3.27–3.48) in females and males, respectively. The excess risk of stroke was mainly attributable to the significantly higher risk of cerebral infarctions with the risk for T2DM being four times that for general population.* Conclusions*. The relationship between stroke and T2DM was strong, especially in female. The incidence of stroke and stroke subtypes among patients with T2DM was up to 3-fold higher than in general population in Zhejiang province, especially the subtype of cerebral infarctions.

## 1. Introduction

Stroke has been recognized as a major problem for public health worldwide. As World Health Organization reported, stroke ranks third (after MI and cancer) as a cause of death around the world [1]. Although stroke incidence has declined in industrialized countries [2], it has increased among urban [3] and rural [4] residents in China over past decades. The prevalence of type 2 diabetes mellitus (T2DM) in Chinese adults has increased faster, too [5]. In Western population, many prospective studies have shown that, compared to population without diabetes, the incidence and mortality of stroke were increased in patients with diabetes [6–9]. The Da Qing IGT and Diabetes Study found that diabetes was associated with a substantially increased risk of death in Chinese adults, especially from CVD, almost half of which was due to stroke [10]. The result of a Chinese hospital-based study showed patients with diabetes presented more frequently with stroke compared with nondiabetics [11]. However, these studies have been performed in selected patients based on hospital. Furthermore, due to studies with small sample sizes, there was insufficient data to describe the stroke incidence in patients with T2DM. To our knowledge, the stroke incidence in patients with T2DM compared to general population is still unknown in Chinese adults, especially population-based. Dependent on Diabetes Surveillance System and Stroke Surveillance System in Zhejiang province, this study aims to use population-based surveillance data to describe the incidence of stroke and stroke subtypes among patients with T2DM.

## 2. Materials and Methods

### 2.1. Data Sources

The long-term surveillance data was from Diabetes Surveillance System and Stroke Surveillance System of Zhejiang Province in China, which was established in 2001 with thirty surveillance districts and covered about 16 million residents. These surveillance districts were selected by geographic variations and socioeconomic status which showed appropriate representativeness for Zhejiang Province [12]. The diabetes and stroke patients were diagnosed by certificated health practitioner and must be reported within a week. Patients' information including demographics, diagnosis, and diagnostic basis was registered in the surveillance system. Then the records were transferred to regional Center for Disease Control and Prevention (CDC) for examination and verification, and the eligible records were pooled together to provincial CDC for further verification to make sure the record was the newly diagnosed and did not report before [13].

For stroke, if the interval between first onset and recurrence was more than 28 days, this record needs to report again. Otherwise, this record need not to report. According to the International Classification of Disease 10th revision (ICD-10), stroke patients were divided into four stroke subtypes, including subarachnoid hemorrhage (I60), intracerebral hemorrhages (I61), cerebral infarctions (I63), and unspecified strokes (I64). Classification and registration were completed by professional health practitioner. Furthermore, in the Diabetes Surveillance System, diabetes was divided into type 1, type 2, gestational, or other types of diabetes. Finally, a total of 327,268 T2DM patients and 307,984 stroke patients were collected between January 1, 2007, and December 31, 2013. This study was carried out in accordance with the “Declaration of Helsinki.”

### 2.2. Data Linkage

**Figure 1:**
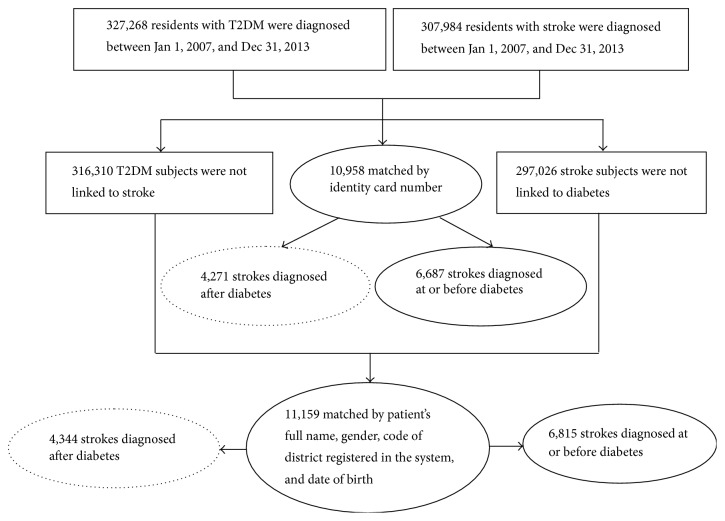
Flowchart of data linkage between Diabetes Surveillance System and Stroke Surveillance System of Zhejiang in China.

In the present study, only the stroke and T2DM patients recorded between January 1, 2007, and December 31, 2013, were included. Two kinds of matching conditions were used to link databases. (1) Identity card number was used to link T2DM patients who suffer stroke in the following years. (2) Patient's full name, gender, code of district registered in the system, and date of birth (year and month) were simultaneously used to link the record which did not match in step one (Figure 1). Those paired records with the date of initial T2DM diagnosis later than stroke were excluded. Finally, 8615 cases of T2DM patients suffering stroke were paired.

### 2.3. Statistical Analysis

The incidence of stroke and stroke subtypes among patients with T2DM was evaluated by standardized incidence ratio (SIR) and 95% confidence interval (CI) comparing with general population. SIR and 95% CI were calculated as the number of observed stroke events divided by the excepted number of events with the Poisson regression model [14]. The numbers of observed stroke events were counted by T2DM patients who suffer stroke in the follow-up years. The excepted number of events was calculated as the number of person-year at risk multiplied by the stroke incidence in general population. The number of person-year at risk was calculated for T2DM patients in both paired and unpaired groups, respectively. For the paired group, person-years were calculated from the data of initial diagnosis of T2DM to the occurrence of stroke. For the unpaired group, person-years were calculated from the data of initial diagnosis of T2DM to the dateline of this study (December 31, 2013). The stroke incidence in general population used stroke incidence of residents in thirty surveillance districts. All analyses were done by using SAS 9.2 (SAS Institute Inc., Cary, NC, USA).

## 3. Results

A total of 327,268 T2DM cases were included from Diabetes Surveillance System between January 1, 2007, and December 31, 2013. During the follow-up time, 8615 stroke events have occurred with 4324 (50.19%) in male and 4291 (48.81%) in female, respectively. The median ages at diagnosis and registration of diabetes were 59 (50, 69) years old and 60 (51, 69) years old, respectively. The baseline characteristics of T2DM and total stroke outcomes were described in Table 1.

**Figure 2:**
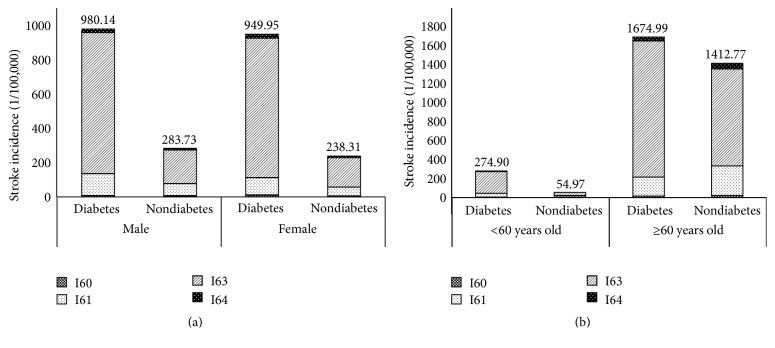
Stroke incidence for the diabetic and nondiabetic populations by age and sex in 2007–2013 in Zhejiang Province. (a) Stroke incidence in male and female. (b) Stroke incidence grouped by age. I60: subarachnoid hemorrhage; I61: intracerebral hemorrhages; I63: cerebral infarctions; and I64: unspecified strokes.

Table 2 showed gender-specific stroke incidence among patients with T2DM and general population. For all patients, the incidence of total stroke among patients with T2DM was 3.60 times compared to general population. For male patients, the SIR for total stroke was 3.38 (95% CI 3.27–3.48). Four subtypes all showed significant increased SIRs for patients with T2DM compared to general population. Cerebral infarctions (I63) had highest SIR compared to other subtypes as 4.08 (95% CI 3.95–4.22). For female patients, the SIR for total stroke was slightly higher than male as 3.87 (95% CI 3.76–3.99). The SIRs increased significantly among four subtypes, especially in cerebral infarctions (I63) (4.60, 95% CI 4.45–4.75). The incidence of total stroke for the T2DM and general population was higher in male compared with female. However, the SIRs in female were higher than male in total stroke. In addition, the stroke incidence per 100,000 person-years for the diabetic and nondiabetic population in male was higher than female as 980.14 and 283.73, respectively (Figure 2).

Table 3 showed the stroke incidence in patients with T2DM and general population between urban and rural area. For urban and rural area, the SIRs for total stroke were 3.36 (95% CI 3.25–3.48) and 3.77 (95% CI 3.67–3.87), respectively. Four subtypes all showed significant increased SIRs for patients with T2DM. The highest SIRs were in the group of cerebral infarctions (I63) which was 3.98 in urban and 4.57 in rural. The stroke incidence and SIRs in rural area were all higher than in urban area.

According to the age at diagnosis of T2DM, the result of overall SIRs for stroke in patients with T2DM was shown in Table 4. Significant difference was detected for total stroke by age. The incidence of strokes among the population over 60 years old was much higher than the population less than 60 years old. However, the condition of SIR was opposite. In the group of <60 years old, T2DM patients suffer increased risk of stroke compared to the general population except subarachnoid hemorrhage (I60). In the group of ≥60 years old, the SIR increased significantly only in cerebral infarctions (I63) with 1.37 times higher than in general population.

## 4. Discussion

This study aimed to explore the subsequent stroke incidence in patients with T2DM based on the long-term surveillance data in Zhejiang province, China. The results showed that the stroke incidence in patients with T2DM was significantly higher than in general population, which were consistent with previous studies [7, 15–17].

The risk for stroke among patients with T2DM was up to 3-fold higher than in general population in male and female, which were comparable to those in large prospective studies [6, 18]. The results of United Kingdom Prospective Diabetes Study (UKPDS) showed that the risk of stroke incidence in patients with T2DM in male was 1.63 times more than in female [19]. The Renfrew/Paisley Study in Scotland [16] and a meta-analysis of 64 cohorts [20] showed that the excess risk of stroke associated with diabetes is significantly higher in female than in male. In the current study, the SIR in female with T2DM was higher than in male in total stroke and all four subtypes. This revealed that, compared to general female population, female with T2DM might have higher risk for stroke. However, the incidence of stroke among general population was higher in male than in female, which was consistent with the result of Sino-MONICA-Beijing Project [3].

The incidence of stroke in rural areas among general population in this study was lower than Tianjin Brain study during 2006–2012 [4] while in urban areas it was higher than Bin Jiang's study during 1991–2000 [21]. It is lacking related study focus on the incidence of stroke in patients with T2DM in China stratified by urbanization level. The risk of stroke in patients with T2DM living in rural areas was 3.77 times than in general population and 3.36 times in urban areas. SIR in rural was higher than in urban areas which was the same as the relationship of stroke incidence among general population between rural and urban area. The reason might be that metabolic control was worse in the rural area in Zhejiang province [22].

Bell found that the relative risk of stroke in patients with T2DM reached a maximum in the 40- to 60-year-old group than in the nondiabetic population [23]. In addition, Kissela et al. found that diabetes is clearly one of the most important risk factors for ischemic stroke, especially in those patients who are less than 65 years of age [24]. This study found that SIR of T2DM patients' age at diagnosis of T2DM less than 60 years old was significantly higher than that of general population. This result was comparable to previous study. However, it was on the contrary to the stroke incidence in general population. It suggested that it was necessary to strengthen the management of patients with T2DM from middle age.

There was a thrombotic tendency or at least an imbalance between the haemostatic and thrombosis-protecting system in diabetic patients, which might play a crucial role in the development of stroke, especially cerebral infarctions (I63) [25]. In this study, the risk of I63 in patients with T2DM was up to 4-fold higher than in general population, higher than the found in the Honolulu Heart Program [26]. Previous studies [11, 27, 28] in diabetic patients have shown a decrease prevalence of both I60 and I61 when compared with the general population. In the current study, the risks of I60, I61, and I64 in patient with T2DM were higher than in general population, which were not consistent with previous studies. This might be due to the fact that other risk factors did not adjust in the study, such as hypertension, cholesterol, and smoking. In one word, the results revealed that T2DM patients had higher risk to suffer I63 than the other three stroke subtypes.

There were several strengths for this study. Firstly, it was one of the few studies exploring the stroke incidence in patients with T2DM by SIR in China. Secondly, it was a population-based surveillance study with a large sample of 327,268 T2DM cases and 8615 stroke event outcomes. The most important is that T2DM and stroke patients were diagnosed and reported by certificated health practitioners, and, to further ensure the quality and veracity of data, the related data was verified by regional and provincial CDCs and registered in the surveillance system eventually.

However, there were some limitations in this study. Firstly, we stratified by the variables of gender, urbanization, and age, while other potential confounding factors including hypertension, cholesterol, smoking, obesity status, alcohol consumption, physical activity, and diabetes treatments have not been adjusted in the analysis. Secondly, the follow-up time was not long enough, which restricted our ability to further assess the potential lead time bias and explore the effect of diabetes duration on stroke incidence.

In conclusion, the current study indicated that the incidence of stroke and stroke subtypes among patients with T2DM was up to 3-fold higher than in general population in Zhejiang province, especially the subtype of cerebral infarctions (I63). Compared to males, the SIR was higher in females, although the incidence of stroke for the diabetic in females was lower than in males. Given the rapid growth of diabetes in China, even a small increased stroke risk would have important public health implications at population level. It is urgent and necessary to prevent and control diabetes.

## Figures and Tables

**Table 1:** Characteristics of the T2DM and stroke cases included in study.

	T2DM	Stroke
Total	327,268	8,615
Gender, *N* (%)		
Male	163,819 (50.06)	4,324 (50.19)
Female	163,449 (49.94)	4,291 (48.81)
Area, *N* (%)		
Urban	130,807 (39.97)	3,166 (36.75)
Rural	196,461 (60.03)	5,449 (63.25)
Age at diagnosis, median (*Q*1, *Q*3)	59 (50,69)	72 (64,79)
Age at registration, median (*Q*1, *Q*3)	60 (51,69)	72 (64,79)

**Table 2:** SIRs in male and female with T2DM compared with general population, 2007–2013 (1/100,000 person-years).

Subtypes	Total					Male					Female				
	Diabetes		General population		SIR (95% CI)	Diabetes		General population		SIR (95% CI)	Diabetes		General population		SIR (95% CI)
	*N*	Incidence	*N*	Incidence		*N*	Incidence	*N*	Incidence		*N*	Incidence	*N*	Incidence	
Total	8615	964.84	307806	268.10	3.60 (3.52–3.68)	4324	980.14	169029	290.36	3.38 (3.27–3.48)	4291	949.95	138774	245.20	3.87 (3.76–3.99)
I60	83	9.30	6306	5.49	1.69 (1.36–2.10)	37	8.39	2974	5.11	1.64 (1.19–2.27)	46	10.18	3332	5.89	1.73 (1.30–2.31)
I61	1031	115.47	72332	63.00	1.83 (1.72–1.95)	563	127.62	42674	73.31	1.74 (1.60–1.89)	468	103.61	29658	52.40	1.98 (1.81–2.16)
I63	7309	818.57	217537	189.48	4.32 (4.22–4.42)	3634	823.73	117424	201.72	4.08 (3.95–4.22)	3675	813.58	100110	176.89	4.60 (4.45–4.75)
I64	192	21.50	11631	10.13	2.12 (1.84–2.45)	90	20.40	5957	10.23	1.99 (1.62–2.45)	102	22.58	5674	10.03	2.25 (1.86–2.73)

I60: subarachnoid hemorrhage; I61: intracerebral hemorrhages; I63: cerebral infarctions; I64: unspecified strokes.

**Table 3:** SIRs in urban and rural with T2DM compared with general population, 2007–2013 (1/100,000 person-years).

Subtypes	Urban					Rural				
	Diabetes		General population		SIR (95% CI)	Diabetes		General population		SIR (95% CI)
	*N*	Incidence	*N*	Incidence		*N*	Incidence	*N*	Incidence	
Total	3166	870.00	102779	258.62	3.36 (3.25–3.48)	5449	1030.08	205027	273.13	3.77 (3.67–3.87)
I60	31	8.52	2268	5.71	1.49 (1.05–2.12)	52	9.83	4038	5.38	1.83 (1.39–2.40)
I61	403	110.74	23798	59.88	1.85 (1.68–2.04)	628	118.72	48534	64.65	1.84 (1.70–1.99)
I63	2667	732.88	73269	184.36	3.98 (3.83–4.13)	4642	877.53	144268	192.19	4.57 (4.43–4.70)
I64	65	17.86	3444	8.67	2.06 (1.62–2.63)	127	24.01	8187	10.91	2.20 (1.85–2.62)

I60: subarachnoid hemorrhage; I61: intracerebral hemorrhages; I63: cerebral infarctions; I64: unspecified strokes.

**Table 4:** SIRs in age at diagnosis of T2DM compared with general population, 2007–2013 (1/100,000 person-years).

Subtypes	<60 years old					≥60 years old				
	Diabetes		General population		SIR (95% CI)	Diabetes		General population		SIR (95% CI)
	*N*	Incidence	*N*	Incidence		*N*	Incidence	*N*	Incidence	
Total	1245	274.90	54611	56.16	4.89 (4.63–5.17)	7370	1674.99	253150	1441.32	1.16 (1.14–1.19)
I60	19	4.21	2791	2.87	1.47 (0.94–2.30)	64	15.01	3514	20.01	0.75 (0.58–0.96)
I61	177	39.24	17051	17.53	2.24 (1.93–2.59)	854	199.64	55261	314.63	0.63 (0.59–0.68)
I63	1030	227.64	33710	34.67	6.57 (6.18–6.98)	6279	1433.67	183804	1046.50	1.37 (1.34–1.40)
I64	19	4.22	1059	1.09	3.87 (2.47–6.07)	173	40.56	10571	60.19	0.67 (0.58–0.78)

I60: subarachnoid hemorrhage; I61: intracerebral hemorrhages; I63: cerebral infarctions; I64: unspecified strokes.
